# The Potential of High Resolution Magnetic Resonance Microscopy in the Pathologic Analysis of Resected Breast and Lymph Tissue

**DOI:** 10.1038/srep17435

**Published:** 2015-12-07

**Authors:** Brittany Z. Dashevsky, Timothy D'Alfonso, Elizabeth J. Sutton, Ashley Giambrone, Eric Aronowitz, Elizabeth A. Morris, Krishna Juluru, Douglas J. Ballon

**Affiliations:** 1 Department of Radiology, Weill Cornell Medical College, Cornell University, New York, NY; 2 Department of Pathology, Weill Cornell Medical College, Cornell University, New York, NY.; 3 Department of Healthcare Policy, Weill Cornell Medical College, Cornell University, New York, NY.; 4 Department of Physics, Weill Cornell Medical College, Cornell University, New York, NY.; 5 Department of Radiology, Memorial Sloan, Kettering Cancer Center, New York, NY.

## Abstract

Pathologic evaluation of breast specimens requires a fixation and staining procedure of at least 12 hours duration, delaying diagnosis and post-operative planning. Here we introduce an MRI technique with a custom-designed radiofrequency resonator for imaging breast and lymph tissue with sufficient spatial resolution and speed to guide pathologic interpretation and offer value in clinical decision making. In this study, we demonstrate the ability to image breast and lymphatic tissue using 7.0 Tesla MRI, achieving a spatial resolution of 59 × 59 × 94 μm^3^ with a signal-to-noise ratio of 15–20, in an imaging time of 56 to 70 minutes. These are the first MR images to reveal characteristic pathologic features of both benign and malignant breast and lymph tissue, some of which were discernible by blinded pathologists who had no prior training in high resolution MRI interpretation.

Breast cancer accounts for 41% of all cancers in women^1^. Breast conservation surgery (BCS) is the standard of care for patients with Stage I and Stage II breast cancer, and may even be considered for some locally advanced breast cancers, rather than mastectomy^2^,^3^,^4^. Adjuvant therapy for these patients is determined on a case-by-case basis. After BCS, the margins of the excised specimen are evaluated for the presence of tumor^5^,^6^. Tumor at the inked margin is the most important predictor of local recurrence^7^. Thus, the American Society of Clinical Oncology (ASCO) recommends that surgically inked margins from BCS be negative for malignancy^8^. Should the pathologist determine that a surgical margin of the tissue contains cancerous cells, the patient must undergo a second operation to obtain margins free of tumor. Since pathologic processing and analysis require at least 12 hours, patients do not learn whether their resected specimen is free of tumor for at minimum one day post surgery. Approximately 25% of women have tumor at the surgical margin and must return at a later date for a second operation^8^. There is a need, therefore, for tools that can expedite pathologic interpretation to allow same-day results and intervention.

Clinical MRI is the most sensitive (94%) screening test available for detecting breast cancer, but lacks specificity (26%)^9^. Since radiology and pathology are distinct disciplines and require multiple sequential steps from the initial identification of a suspicious lesion on imaging to pathologic diagnosis, this results in a delay in patient care. After imaging patients must undergo percutaneous biopsy and/or excision of a suspicious lesion to obtain a definitive diagnosis.

MR Microscopy (MRM) generally refers to MRI techniques that achieve sub 100 μm resolution^10^. While this resolution is substantially poorer than that achieved with light microscopy (0.25 μm), it far exceeds that of MRI in standard clinical practice (approximately 1 mm in plane resolution)^3^, allowing visualization of histologic detail.

To date, few studies have evaluated 7.0 Tesla MRI performance in breast^11^,^12^,^13^,^14^,^15^ and lymph tissue^16^,^17^,^18^ and published studies have yet to achieve sufficient resolution to allow pathologic diagnosis on MRI. Therefore, we sought to: (1) develop an MRM technique optimized for imaging resected breast and lymph tissue immediately after excision with a short acquisition time to provide value in clinical decision making, and (2) compare the diagnostic performance of images obtained using MRM to images obtained from light microscopy of traditional hematoxylin & eosin (H&E) stained specimens.

## Results

### MRM technique optimized for imaging breast and lymph tissue within 1–2 hours

**Figure 1:**
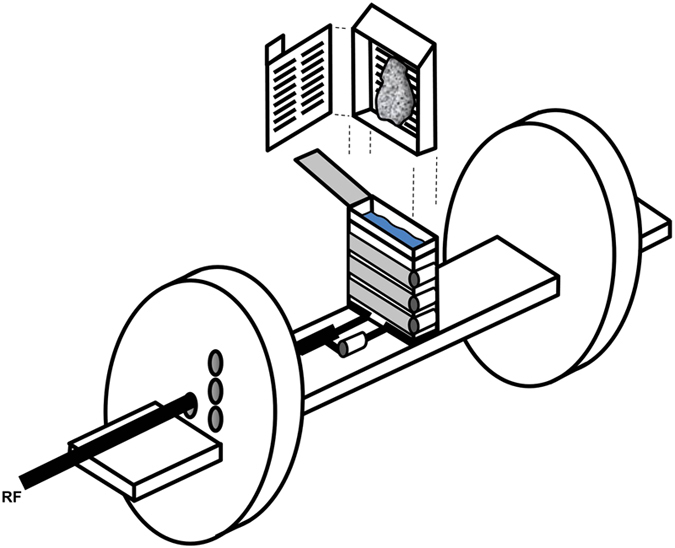
Specimens were obtained at 7.0 Tesla with T_1_ gradient echo sequences using the above custom designed coil/cassette apparatus, which included a three loop parallel wound solenoidal resonator. A Gd-DTPA solution was contained in the reservoir in which the cassette and sample were immersed. The two polyethylene foam discs supporting the platform helped to dampen scanner vibration.

A 7.0 Tesla 30 cm bore magnetic resonance imaging system was optimized for imaging fresh breast and lymph node tissue obtained from lumpectomy and/or axillary lymph node dissection (Fig. 1), to achieve an imaging spatial resolution of 59 × 59 × 94 μm^3^. The technique included a custom designed receptacle for specimens integrated into a solenoidal radiofrequency resonator that greatly facilitated the registration of MRM and light microscopy images. A total of 10 specimens were imaged. The average imaging time per specimen was just over one hour. Pathologies of tissues, as determined by subsequent standard light microscopy assessment, included normal breast parenchyma (n = 3), fibroadenoma (n = 2), ductal carcinoma *in situ* (DCIS) (n = 1), invasive ductal carcinoma (IDC) and DCIS (n = 1), invasive lobular carcinoma (ILC) (n = 1), in addition to benign axillary lymph node (n = 1) and axillary lymph node containing metastatic carcinoma (n = 1).

### MRM of Breast Tissue

**Figure 2:**
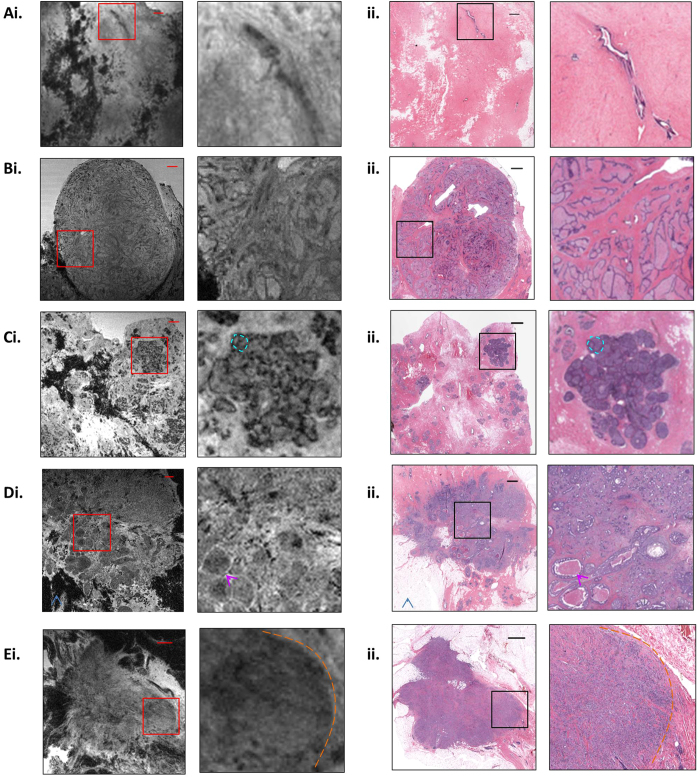
7.0 Tesla MR image (i) and corresponding H&E stained light microscopy image (ii) of normal breast parenchyma (**A**), fibroadenoma (**B**), DCIS (**C**), IDC and DCIS (**D**) and ILC (**E**). An expanded view of the area within the boxes is shown on the right, at 4× magnification. The scale bar at top right is 1.25 mm. A single expanded duct is highlighted in cyan (**C**). Fat surrounding the IDC mass is identified (blue arrowhead; (**D**)), appearing black due to fat suppression on MRI. Central debris within DCIS is highlighted (purple arrowhead; (**D**)). The lateral margin of the ILC mass is delineated in orange (dashed line; (**E**)).

We began the *ex vivo* studies by first evaluating normal breast tissue obtained from patients undergoing elective reduction mammoplasty. This was chosen to confirm that the technique was successful in obtaining adequate resolution of identifiable normal structures, as confirmed by a breast pathologist. Normal breast parenchyma (Fig. 2A) appears as fibroglandular tissue with interspersed globular hypointense fat. Microscopic examination confirmed benign inactive breast glandular tissue in predominantly fibrous stroma.

Fibroadenoma (Fig. 2B) appears as a well-circumscribed ovoid nodular lesion, here measuring up to 2.5 cm in maximum diameter. Internally the fibroadenoma is comprised of a cleft-like network of tissue isointense to surrounding breast parenchyma. On histology, the fibroadenoma is a circumscribed tumor with stromal and glandular components. The tumor shows an intracanalicular pattern, which corresponds to the cleft-like network on MRM. This pattern is characterized by compression of glands by the stroma, forming slit-like spaces.

We next evaluated malignant pathology, including specimens from patients that had biopsy proven carcinoma (Fig. 2C–E). DCIS (Fig. 2C) appears as a cluster of rounded and ovoid structures containing central hypointense material. This corresponds on histology to expanded ducts and lobules containing a solid proliferation of monotonous neoplastic cells of intermediate nuclear grade. Luminal necrosis is evident in some ducts.

The specimen which contained both IDC and DCIS (Fig. 2D) had an area of centrally expanded ducts which was both visually similar to the MRM DCIS specimen and corresponded microscopically to areas of micropapillary and cribriform DCIS with luminal necrosis (box). These ducts were present within an enhancing irregular mass, which corresponded to IDC on microscopic examination. MRM demonstrates wisp-like linear hyperintense striations extending peripherally from the tumor margin. On histology, these findings correspond to a 1.6 cm well-differentiated IDC characterized by tubules growing haphazardly within a desmoplastic stroma and infiltrating the adjacent tissue.

ILC (Fig. 2E) appears as an enhancing, irregular mass absent of gland formation in the background of normal parenchyma. On histology, ILC was characterized by tumor cells growing in single file arrangement and as solid nests into adjacent breast and adipose tissue. Cells were of intermediate nuclear grade. An E-cadherin immunostain (not shown) demonstrated absent membranous reactivity in tumor cells, confirming a diagnosis of ILC.

### MRM of lymph nodes

**Figure 3:**
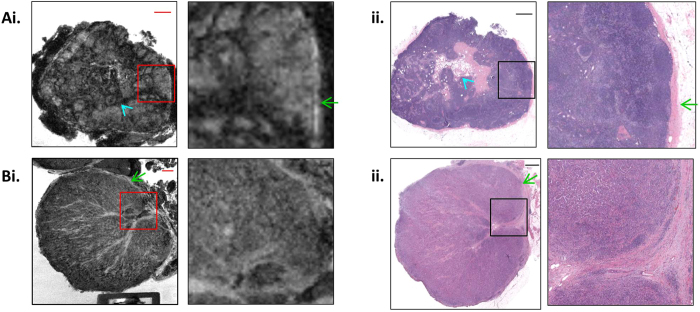
7.0 Tesla MR image (i) and corresponding H&E stained light microscopy image (ii) of a benign lymph node (**A**) and a lymph node with carcinoma invasion (**B**). An expanded view of the area within the box is demonstrated on the right, at 4× magnification. The scale bar at top right is 1.25 mm. The lymph node capsule is highlighted (green arrow; (**A,B**)). Normal lymph node hilum is highlighted (cyan arrowhead; (**A**)).

We next imaged benign and malignant axillary lymph nodes (Fig. 3A,B). A normal lymph node (Fig. 3A) contains rounded, hyperintense areas around its periphery with lower central signal intensity, corresponding on histology to lymphoid follicles surrounding a central mixed-attenuation fatty/fibrous hilum. The lymph node shown here measures 1 cm in maximum diameter and is bounded by a 120 μm thick smooth capsule. This capsule is undisrupted in its imaged course. This lymph node was negative for metastatic carcinoma on H&E examination. A negative cytokeratin (AE1/AE3) immunostain excluded the presence of micrometastatic deposits and isolated tumor cells.

In contrast, a lymph node containing metastatic carcinoma (Fig. 3B) exhibited replacement of the normal lymphoid architecture with a disorganized matrix. Small segments of normal capsule are visualized, with the remaining capsule disrupted by tumor invasion. On histology this lymph node was virtually replaced by metastatic poorly-differentiated ductal carcinoma. Extracapsular extension was not present.

### Comparison of Diagnostic Performance of MRM to Light Microscopy

**Figure 4:**
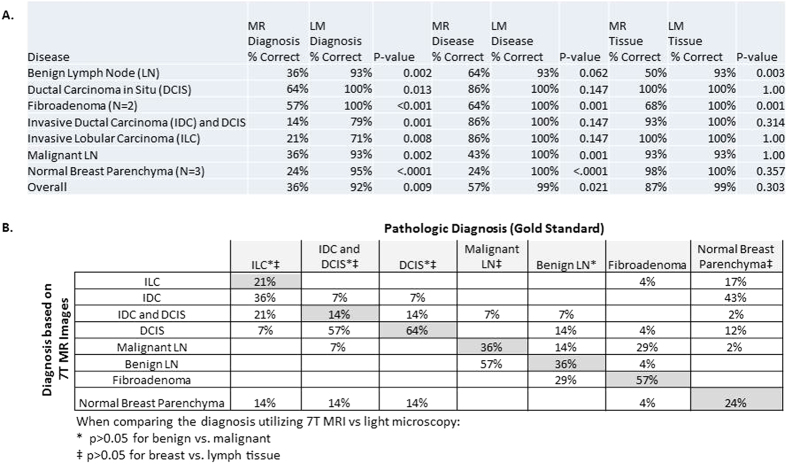
Survey results of the diagnosis, disease (benign vs. malignant) and tissue type (breast vs. lymphatic) utilizing representative 7.0 Tesla MR images of each specimen, in comparison to results for the corresponding light microscopy image (A). The individual responses for each MR image are depicted in (**B**), with the correct diagnosis highlighted in gray.

Utilizing MRM, fourteen pathologists selected the correct diagnosis (exact diagnosis) for 36% of cases, the correct disease process (benign vs. malignant) for 57% of cases, and the correct tissue type (breast vs. lymph node) for 87% of cases, compared to 92%, 99% and 99% respectively, utilizing light microscopy images of H&E stained specimens (Fig. 4A). The results were significantly different for 7.0 Tesla MR versus light microscopy in determining the correct diagnosis (p = 0.01) and disease process (p = 0.02). There was no significant difference between the two modalities in selecting tissue type (p = 0.30). Though the pathologists were unable to identify many scanned specimens, more than half of pathologists correctly identified DCIS and fibroadenoma specimens. In addition, pathologists correctly selected a malignant versus benign disease, with no significant difference between MR and light microscopy for the following specimens: benign lymph node, DCIS, IDC and DCIS, and ILC. Figure 4B illustrates pathologists’ individual responses to the MR images for each pathologic diagnosis.

## Discussion

This study demonstrates the successful development of a process involving tissue preparation and a custom-built specimen chamber and resonator to obtain *ex-vivo* images of breast and lymph tissue at 7.0 Tesla with a spatial resolution that is 1–2 orders of magnitude better than conventional breast MRI. Slice-matched MRM and light microscopy images demonstrated numerous structural similarities in both benign and malignant specimens. Glandular tissue, ducts, tubules, lymph follicles, and tissue margins were all apparent when MRM images were viewed alongside their slice-matched light microscopy counterparts. Whereas the light microscopy images took over 12 hours to prepare, MRM images could be obtained in approximately 1 hour.

The structural similarities with light microscopy, in combination with a significant decrease in time needed to obtain images, introduces a unique role for MRI in the management of breast cancer. First, MRM has the potential to guide intra-operative decision making. Second, it may serve as a tool for pathologic navigation.

Traditional pathologic assessment of surgical specimens is limited by processing time. Pathologic margin status can only be determined post-operatively, and because of the long histopathologic evaluation time, a diagnosis of positive tumor margins requires another surgery for re-excision. A more expedited determination of surgical margins could guide the intra-operative decision to remove additional tissue at the time of initial surgery for patients with positive margins, thereby eliminating the necessity of a second procedure, reducing patient discomfort and anxiety, reducing medical costs, and potentially reducing surgical morbidity.

While intra-operative MRI is not routinely utilized for evaluation of breast conservation margins, it is used routinely at some institutions to assist with resection of brain tumors, such as glioblastoma multiforme. In the intra-operative setting, the surgical suite must be prepped, patient repositioned and utensils recounted to ensure MR safety prior to imaging. Intraoperative scan time then takes at minimum 20 additional minutes, during which time the operation is on hold. Our current 57 to 74 minute specimen scan time is slightly longer, but offers other advantages. Specimen MRI is much cheaper, safer and allows the surgery to proceed while the specimen is being imaged. For example, in the case of BCS, specimen margins may be analyzed while the surgeon is performing a sentinel lymph node dissection.

Ideally, the specimen imaging time would be further shortened to allow for a more comprehensive pathologic analysis during the intra-operative period. Our scan time was chosen to test proof-of-principle at a spatial resolution of 59 × 59 × 94 μm^3^, but shorter scan times are possible if reductions in the spatial resolution or signal-to-noise ratio can be tolerated. For example, a reduction from 60 to 30 minute scan time at a spatial resolution of 70 × 70 × 94 μm^3^ can be performed with no loss in signal-to-noise ratio. Alternatively, the small tissue sample size suggests that the incorporation of cryoprobe technology might improve the sensitivity of the method by a factor of two or more.

Currently, only a small portion of a tissue specimen is selected for pathologic tumor grading, which may cause areas of tumor to be entirely missed or graded inaccurately. Traditionally the pathologist palpates the entire resected specimen, identifies an area that is felt to represent a mass, and performs a slice through that area for microscopic analysis. The specimen is then further assessed by slicing at regular intervals. With MRM, 3D images can first be obtained through an entire resected specimen without physically slicing the tissue. A video of the gradient echo T_1_ acquisition of the fibroadenoma specimen shown in Fig. 2B (See Supplementary Information) demonstrates the 3D capabilities of 7T MRI. Although images are of a lower resolution than light microscopy, this may serve as a useful navigational tool to guide a pathologist to areas of interest within a resected specimen and improve the diagnostic yield.

While not the primary goal of this work, in order to test the diagnostic accuracy of MRM in evaluation of breast and lymph tissue, images were presented to blinded pathologists with no prior experience in MRM interpretation. There was wide variability in rendering both an exact diagnosis and also in the simpler task of differentiating benign from malignant tissue (Fig. 4A). The pathologists’ accuracy in evaluation of light microscopy images, on the other hand, was close to 100%, except for the ILC image, probably due to lack of imaging with E-cadherin staining, which is traditionally used for ILC diagnosis. These results are not surprising. MR and light microscopy images differ not only in spatial resolution, but also in the presence and meaning of color, grayscale, and brightness. Despite this lack of training, the pathologists did better than expected in several areas. For example, when presented with MRM images of DCIS, pathologists made the correct diagnosis in 64% of cases, and properly identified the tissue as malignant in 86% of cases (Fig. 4A,B). When presented with MRM images of ILC, pathologists made the correct diagnosis in only 21% of cases, but properly identified the tissue as malignant in 86% of cases. This suggests that there are architectural similarities between the MRM and LM images.

If MRM is to be used for intra-operative decision making or as a tool for pathologic navigation, then differentiation of benign from malignant tissue is the primary objective, allowing for an exact diagnosis to be made at a later time with light microscopy. Our results, therefore, show promise that MRM may serve these intended roles. In addition, at this early stage there are no experts in interpreting MR images at the microscopic level, either within the disciplines of pathology or radiology. Successful clinical interpretation of MRM will require contributions from both fields. It is reasonable to expect that with appropriate training in the interpretation of MRM, rendering a diagnosis will be much more accurate than we demonstrated in our study.

Prior work on *in vitro* MRM of the breast is limited. Cebulla *et al.* reported *ex vivo* imaging of breast cancer xenograft angiogenesis with spatial resolution reaching 40 μm on a 9.4 Tesla spectrometer and 8 μm in microCT^19^. Their work evaluated blood vessels in the context of tumor angiogenesis and used overnight Gd-DTPA marination. With 9.4 Tesla MRI Abe *et al.* were able to evaluate tumor margins of invasive breast cancers in patients who received intravenous contrast immediately prior to resection at 200–470 μm in plane resolution^20^. Multiple studies have demonstrated retention of contrast in both breast cancers^16^ and lymph nodes with malignant invasion^17^ when intravenous contrast is administered prior to excision.

Other work performed *in vivo* has demonstrated that breast imaging is feasible at 7.0 Tesla utilizing a custom-designed radiofrequency coil and pulse sequences with isotropic resolution ranging from 450 to 700 μm^11^,^12^,^14^. At this resolution, however, visualization of important architectural features of tissue is limited and post excision pathologic analysis is still required. With our high resolution *ex vivo* technique for the first time we are able to visualize distinctive features of both benign and malignant lesions, making pathologic diagnosis within reach. Our short acquisition time also results in increased clinical utility.

The main limitation of this study is that participating pathologists had no prior experience in interpreting MR images, thus their interpretation of 7.0 Tesla MR images simply demonstrates the variable degree of similarity to traditional light microscopy images of H&E stained specimens. Also, while it was possible to demonstrate excellent spatial correspondence between MRM and light microscopy in a given specimen, the pathologic sample consisted of only a limited number of slices, as is typical, and thus prevented a whole-specimen comparative analysis.

In conclusion, MRM can be optimized to achieve high resolution images of resected breast and lymph tissue that may be used to differentiate benign from malignant pathology. These images can be obtained in just over an hour. This technique offers the possibility of providing valuable information to surgeons in the intra-operative setting, and may serve as a navigation tool to help pathologists localize areas of concern within resected tissue, thereby reducing sampling error. Our technique can readily be extended to analysis of a variety of tissues other than breast. Importantly, our report describes a potential paradigm shift in the standard-of-care analysis of surgical breast specimens whereby radiology and pathology are merging fields.

## Methods

### Regulatory Requirements

Human breast and lymph tissue was obtained from subjects who were undergoing breast reduction or excision as part of their clinical care (N = 10). All subjects signed informed consent under a protocol approved by the Institutional Review Board at Weill Cornell Medical College.

### Specimen Preparation and Handling

Resected tissue was transported from the operating room to the institutional pathology laboratory per routine procedure. Fresh specimens were sliced in 3–4 mm sections and a single slice was placed into a standard pathology cassette with inside dimensions of 30 × 27 × 5 mm. The specimen containing cassette was hand delivered to our Imaging Center within 30 minutes of removal from the subject. The cassette containing specimen was immersed in 1% gadolinium-diethylenetriaminepentaacetic acid (Gd-DTPA) and 0.9% saline solution immediately prior to imaging and remained immersed for the duration of imaging. After imaging, the specimen was transported to the pathology department for histological evaluation. The total time from surgical resection to return of the sample did not exceed three hours, and typically we obtained useful images in approximately 1 hour.

### MRM at 7.0 Tesla

To improve specimen resolution, an in-house custom-built three turn parallel wound solenoidal transmit/receive resonator was designed to allow imaging of surgically excised breast specimens within a pathology cassette (Fig. 1).

Images were acquired on a Bruker Biospec 30 cm bore magnetic resonance imaging system equipped with B-GA 12S gradient coils rated at 450 mT/m. The custom-built 7.0 Tesla transmit/receive resonator was constructed consisting of a pine platform isolated from the magnet bore by polyethylene foam cylinders at either end. Upon the platform a clear acrylic container was mounted upright. The resonator was fixed to the container via adhesive backing on the copper foil comprising the loops. The container was designed to accept the plastic pathology cassette snugly. We previously employed a similar technique in evaluation of *ex vivo* prostatectomy specimens^21^.

Gradient echo T_1_ acquisition parameters were applied with a field-of-view = 60 × 60 × 12 mm^3^, effective echo time = 13.1 ms, sequence repetition time = 26.3 s, matrix size = 1024 × 1024 × 128 yielding a slice thickness = 94 μm. A total of 2 or 3 sequential 57 minute acquisitions were acquired for each specimen. Utilizing 3–4 mm thick specimens we achieved a resolution of 59 × 59 × 94 μm^3^ with signal-to-noise ratios of approximately 15–20 in each 57 minute acquisition. For specimens with high fat content, fat suppressed gradient echo T_1_ weighted sequences were obtained (scan time: 70 minutes).

A total of 10 specimens were imaged. Pathologies of tissues were subsequently determined by standard light microscopy assessment, and included normal breast parenchyma (n = 3), fibroadenoma (n = 2), DCIS (n = 1), IDC and DCIS (n = 1), ILC (n = 1), in addition to benign axillary lymph node (n = 1) and axillary lymph node containing metastatic carcinoma (n = 1).

The cassette system developed and optimized for sectioned specimens fixed each sample in position, thereby limiting motion or vibration due to rapidly switching gradients, helping attain high resolution images. Also, since the cassette aligned the specimen spatially for MRI, subsequent slicing of the specimen from the cassette rendered straightforward registering of MRM and pathology images.

### Histologic Preparation and Examination

Following completion of MRI, the specimens were returned to the pathology department, where they were fixed in formalin and processed routinely. Five-μm thick hematoxylin and eosin (H&E) stained sections were prepared from paraffin blocks and scanned on an Aperio Scan Scope AT and viewed on Aperio Image Scope (Leica Biosystems, Buffalo Grove, IL, USA). MRM and light microscopy images were compared.

### MRM/Pathology Survey

An online in-house reader study was prepared, whereby 14 pathologists (10 attending physicians, 3 residents and 1 fellow) at NewYork Presbyterian hospital assessed the diagnostic potential of 7.0 Tesla MRM. The untrained blinded readers were asked to select the correct diagnosis for 10 specimens imaged utilizing 7.0 Tesla MRI, followed by the slice matched light microscopy images, arranged in random order. No pathologist had any prior experience with MRI or MRM.

### Statistical Analysis

To analyze survey responses for MR and pathology, the proportion of correctly identified images for specific disease, as well as disease status (benign versus malignant) and tissue type (breast or lymph tissue) were calculated. Furthermore, the overall average percent correctly identified for each specific diagnosis, as well as disease and tissue type were calculated. Differences between MRM and pathology images for each survey question and the overall percent correctly identified were calculated using a two-sample proportion test. All p-values less than 0.05 were considered statistically significant. All analyses were performed using SAS v9.3 (SAS Institute, Cary, NC).

## Additional Information

**How to cite this article**: Dashevsky, B. Z. *et al.* The Potential of High Resolution Magnetic Resonance Microscopy in the Pathologic Analysis of Resected Breast and Lymph Tissue. *Sci. Rep.* **5**, 17435; doi: 10.1038/srep17435 (2015).

## Supplementary Material

Supplementary Video
